# Genotyping of *Helicobacter pylori* Virulence Genes *cagA* and *vacA*: Regional and National Study

**DOI:** 10.1155/2021/5540560

**Published:** 2021-06-29

**Authors:** Rania M. Kishk, Nashaat M. Soliman, Maha M. Anani, Nader Nemr, Ayman Salem, Fawzy Attia, Amal Nooredeen Ahmed Allithy, Marwa Fouad

**Affiliations:** ^1^Microbiology and Immunology Department, Faculty of Medicine, Suez Canal University, Ismailia, Egypt; ^2^Endemic and Infectious Diseases Department, Faculty of Medicine, Suez Canal University, Ismailia, Egypt; ^3^Clinical Pathology Department, Faculty of Medicine, Suez Canal University, Ismailia, Egypt; ^4^Internal Medicine Department, Faculty of Medicine, Suez Canal University, Ismailia, Egypt; ^5^Pathology Department, Faculty of Medicine, Sohag University, Sohag, Egypt

## Abstract

*Helicobacter pylori* (*H. pylori*) plays a crucial role in the pathogenesis of gastritis, peptic ulcer, and gastric cancer. The presence of pathogenicity islands (PAI) genes contributes to the pathogenesis of many gastrointestinal disorders. Cytotoxin-associated gene A (*cagA*) and vacuolating cytotoxin gene (*vacA*) are the most known virulence genes in *H. pylori*. So, our aim was to study *H. pylori* virulence genes' role in gastric disorders pathogenesis. Our study included 150 adult patients who suffered dyspeptic symptoms and were referred to the GIT endoscopy unit. Gastric biopsies were attained for rapid urease test (RUT) and histopathological examination, and multiplex PCR technique for detection of virulence genes was performed. It was found that 100 specimens were (RUT) positive, of which sixty samples (60%) were PCR positive for *H. pylori ureC* gene. The *vacA* and *cagA* genes were identified in 61.6% and 53% of *H. pylori* strains, respectively. Only 5 cases were *vacA*-positive and *cagA*-negative. The most virulent *vacA* s1 allele existed in 56.6% of cases. Out of the 60 *H. pylori* strains, 66% had at least one virulence gene and 34% did not show any virulence gene. *H. pylori* infection showed significant increase with age. *H. pylori* are prevalent amid dyspeptic patients in our region. The main genotype combinations were *vacA*+/*cagA*+ of *s1m1* genotype and they were frequently associated with peptic ulcer diseases, gastritis, and gastroesophageal reflux disease.

## 1. Introduction

*H. pylori* infection causes Peptic Ulcer Disease (PUD) and Gastric Carcinoma (GC) and affects almost half of the world's population [1]. *H*. *pylori* virulent strains and the host genetic condition were blamed for a wide variety of gastric disorders. *H. pylori* virulence genes encode the proteins which are responsible for damaging the gastric epithelium.

The *H. pylori* pathogenicity island (PAI) was initially known as cytotoxin-associated gene (*cag*), since it was assumed to be related to expression of the vacuolating toxin (*vacA*). However, it was afterwards demonstrated that both factors, *vacA* and the PAI, are separate of each other, even though *cag*-negative strains often do not express *vacA* [2].

The pathogenicity island (*cagA*-PAI) is found in the most virulent *H. pylori* strains. *CagA*-PAI is about 40 kb region, containing 31 genes that encode a type IV secretion system, related to *cagA* translocation and the host's inflammatory response [3]. *Cag*A encodes a 120–145 kDa immunodominant protein and is placed at the end of the *cagA*-PAI [4]. This protein is located on the plasma membrane and is phosphorylated at Glu-Pro-Ile-Tyr-Ala (EPIYA) motifs [5].

The numbers and types of EPIYA motifs at the C-terminal region determine the biological activity of *cagA*. After translocation, *cagA* disrupts the signal transduction of gastric epithelial cells producing cytokines which cause chronic gastritis and induce carcinogenesis designating *cagA* as the first bacterial oncoprotein [6].

Vacuolating cytotoxin A (*vacA*) can interrupt the endocytic trafficking, release organic anions and HCO_3_, enhance the immune tolerance, and promote chronic infection by inhibiting immune cells, stimulation of protein kinases, and autophagy modulation [7]. *H. pylori* strains carrying the *vacA* gene vary in their vacuolating ability, due to five *vacA* regions variations: s-region (*s*1 and *s*2), i-region (*i*1, *i*2, *i*3), m-region (*m*1 and *m*2), d-region (*d*1 and *d*2), and c-region (*c*1 and *c*2) [8].

*VacA* gene m region variants affect toxin binding to host cells [9]. *vacA s*1*m*1 variants are the most virulent arrangement, *s*1*m*2 strains toxin production is modest, and *s*2*m*2 strains are rare or not found [10].

Although it is attained in childhood, the highest age groups at risk of *H. pylori* infection and its mode of transmission are unclear [11]. Identifying the age at which infection may cause gastric symptoms in Egyptian patients would help to appropriately apply preventive plans.

The application of multiplex PCR methods improved our information about *H. pylori* regulatory genes. In this work, we studied *H. pylori* virulence genes in infected patients suffering from dyspepsia after gastric biopsy and the association between those genes and endoscopic findings detected.

## 2. Materials and Methods

This cross-sectional descriptive study involved 150 adult dyspeptic patients who were referred to GIT endoscopy unit, aged between 18 and 75 years. Patients with previous gastric surgery were excluded from the study. Patients receiving antimicrobial agents, bismuth, proton pump inhibitors, or H2 receptor antagonists within the 4 weeks preceding endoscopic examination were also excluded. Informed consents were taken from all participants before starting our study. The ethics committee had reviewed and approved the study.

From each patient enrolled in our study, three gastric biopsies were obtained (two antrum biopsies and one fundus biopsy). One of them was used to screen *H. pylori* positive specimens by rapid urease test (RUT). Other biopsies were transported to the pathology lab for histopathological evaluation according to the revised Sydney classification for gastritis [12], using both H&E staining and Giemsa stain, and according to the microbiology lab in brucella broth with 0.5% agar as a transport media for polymerase chain reaction (PCR) technique.

In patients with RUT positive, DNA from gastric biopsies was extracted, after being crushed and homogenized well, using QIAamp DNA Mini Kit (50) 51304 from QIAGEN, USA (catalogue number #51304) following the manufacturer's instructions. First, the samples were incubated at 37°C for 12 h in 2 ml brucella broth media and then centrifuged at 10.000 ×g for 5 min. PCR was performed to amplify the *ureC* (*glmM*) gene (nt 784–1077, 294 bp) using the following primers: forward primer: 5′-AAGCTTTTA GGGGTGTTAGGGGTTT-3′ and reverse primer: 5′-AAGC TTACTTTCTAACACTAACGC-3′. PCR conditions consisted of 30 amplification cycles (1 min at 93°C, 1 min at 55°C, and 1 min at 72°C) [13].

Multiplex PCR was used to identify *vacA* and *cagA* genes in samples positive for *ureC* (*glmM*) gene [14] using primers for *vacA s*1*/vacA s*2*, vacA m*1*/vacA m*2, and *cagA* (Table 1). Agarose gel (2%) with ethidium bromide was used for separation of the PCR product, using a 100 bp ladder as DNA molecular weight standard.

### 2.1. Statistical Analysis

Statistical analysis was done using IBM Statistical Package for the Social Sciences (SPSS) software, version 20.0, for Windows. Data was presented as mean ± standard deviation or percentages. Chi-squared test was used for categorical variables. *P* value was significant at < 0.05.

## 3. Results

150 patients were enrolled from the endoscopy unit. Age ranged from 18 to 75 years (57.2 ± 16.34); 60% of them were females, and 65% of them were from rural area. No one received nonsteroidal anti-inflammatory drugs. Recurrent abdominal pain was the most common presentation followed by nausea, vomiting, dyspepsia, and heart burn (60%, 39%, 24%, and 20%, respectively).

**Figure 1:**
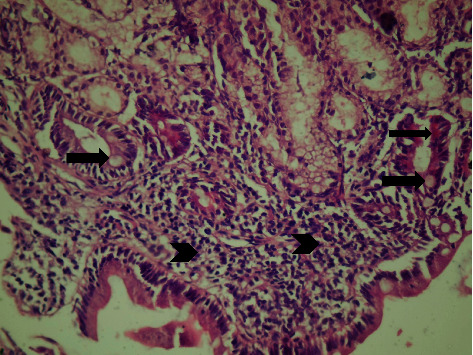
Photomicrograph showing antral chronic superficial gastritis with mixed inflammatory cell infiltrate (arrow heads), mainly lymphocytes and plasma cells; some glandular epithelial cells show intestinal metaplasia, with goblet cells (thick arrow) and Paneth cells (thin arrows), H&E, ×200.

**Figure 2:**
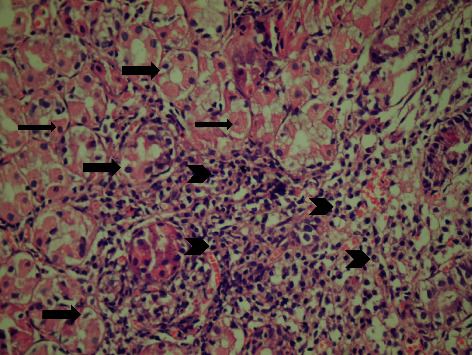
Photomicrograph showing chronic superficial gastritis with mixed inflammatory cell infiltrate (arrow heads), mainly lymphocytes and plasma cells; some glandular epithelial cell shows reactive changes, for example, subnuclear vacuolization (arrows) and parietal cell hyperplasia (thin arrow), H&E, ×200.

**Figure 3:**
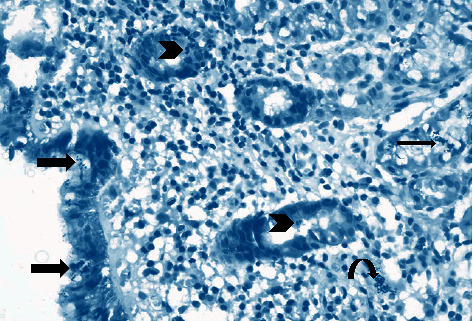
Photomicrograph showing chronic superficial active gastritis (arrow heads pointing to neutrophils attacking glandular epithelium), with mixed inflammatory cell infiltrate, mainly lymphocytes and plasma cells. *Helicobacter pylori* are present in the superficial mucous layer (thick arrow), within the glands, and in the lamina propria (curved arrow), Giemsa stain, ×200.

**Figure 4:**
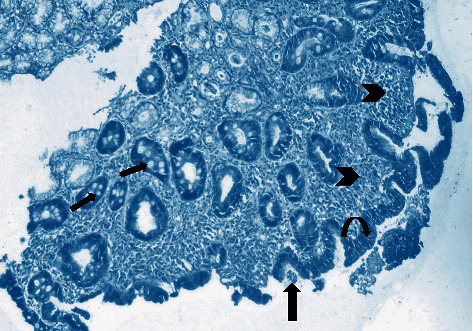
Photomicrograph showing atrophic gastritis with mixed inflammatory cell infiltrate (arrow heads). *Helicobacter pylori* are present in the superficial mucous layer (thick arrow) and intestinal metaplasia with goblet cells (thin arrow), with marked loss of mucosal glands and mucin depletion in the surface epithelium due to regenerative changes (curved arrow). Giemsa stain, ×100.

**Figure 5:**
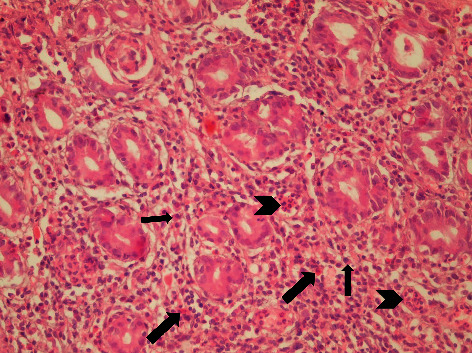
Photomicrograph showing chronic active gastritis with large number of neutrophils (arrow heads) and mixed inflammatory cell infiltrate, namely, plasma cells (thin arrow), lymphocytes (arrow heads), and eosinophils (thick arrow), with moderate atrophy, H&E stain, ×200.

**Figure 6:**
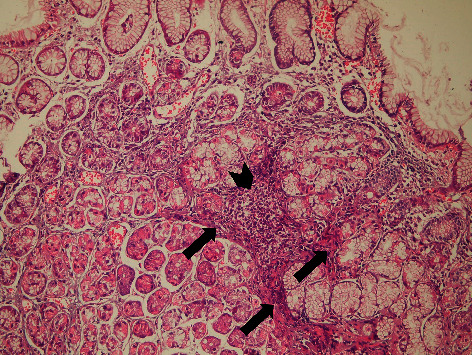
Photomicrograph showing chronic gastritis with mixed inflammatory cell infiltrate and lymphoid follicle formation (arrow head). There is also intramucosal fibrosis (arrows) with moderate glandular atrophy, H&E stain, ×100.

Antral gastritis was the most predominant endoscopic finding followed by Gastroesophageal Reflux Disease (GERD) and pangastritis (36.6%, 34%, and 26.6%, respectively). Normal gastric mucosa was found in only 4 patients (2.6%). Chronic superficial gastritis was the most common finding in histopathological examination, some of which showing mild chronic inflammation with no activity (Figure 1) and others showing moderate chronic inflammation (Figures 2 and 3), followed by atrophic gastritis (Figures 4 and 5), chronic active gastritis (Figure 6), and duodenitis (70%, 20%, 10%, and 6%, respectively).

Out of the 150 studied patients, 100 patients (66.6%) were RUT positive. All patients with antral gastritis and combined duodenal and gastric ulcers were RUT positive. Antral gastritis, diffuse erosive gastroduodenitis, duodenal ulcer, gastric ulcer, and combined duodenal and gastric ulcer had statistically significant association with RUT (*P* < 0.001, <0.001, 0.03, 0.017, and 0.04, respectively).

**Figure 7:**
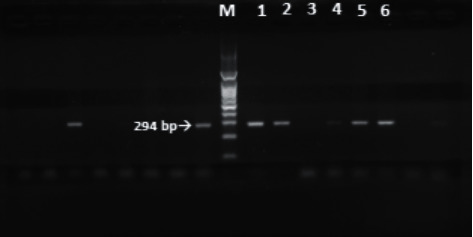
PCR amplification of *UreC* (*glmM*) for *H. pylori* detection: M: 100 bp ladder; lanes 1, 2, and 4–6: positive (at 294 bp); lane 3: negative.

PCR was performed for all RUT positive samples (100 samples), where 60 samples (60%) were positive for *H. pylori ureC* (*glmM*) gene (Figure 7). Antral gastritis represented the most common endoscopic result in patients positive for *H. pylori* by PCR followed by duodenal ulcer and GERD (33.3%, 28.3%, and 25%, respectively). Pan gastritis, diffuse erosive gastroduodenitis, duodenal ulcer, gastric ulcer, and combined duodenal and gastric ulcer showed statistically significant relation with PCR results (*P* < 0.001) (Table 2).

**Figure 8:**
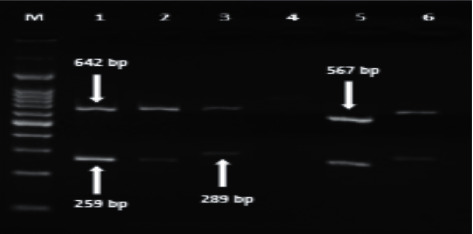
PCR amplification of *vacA* alleles and *cagA:* M: 100 bp ladder; lanes 1–3, 5, and 6: *vacA*+/*cagA*−; lane 4: *vacA*−/*cagA*−; lane 1: *s*1*m*2 genotype; lane 2: *s*1*m*2 genotype; lane 3: *s*2*m*2 genotype; lane 5: *s*1*m*1 genotype; lane 6: *s*2*m*2 genotype.

*vacA* and *cagA* genotypes were identified by multiplex PCR (Figure 8). The *vacA* and *cagA* genes were identified in 37 (61.6%) and 32 (53%) of the 60 *H. pylori* strains, respectively. All strains with *cagA* gene were *vacA* gene positive. Only 5 cases were *vacA* positive and *cagA* negative.

*H. pylori* patients with PUD, gastritis, and GERD (50%, 41.6%, and 25%, respectively) had statistically significant association with *cagA* genotype (*P* < 0.001). The most virulent *vacA* s1 allele was represented in 34 cases (56.6%). The middle region *vacA m*1 was predominant in 27 cases (45%), while *m*2 and *s*2 genotypes were detected in 10 (16.6%) and 3 (5%) cases, respectively (Table 3).

The *vacA s*1*m*1/*cagA*+ was found in 26 cases (43.3%), while *vacA s*1*m*2/*cagA*+ were found in 5 strains only (8.3%). We did not find *vac s*2*m*1 genotype, either *cagA* positive or negative. The *vac s*2*m*2 genotype was detected in 3 patients; one of them was *cagA* positive. Out of the 60 *H. pylori* strains tested, 39 (66%) had at least one virulence gene and 21 (34%) did not show any virulence genes (Table 3).

The age of the patients positive for *H. pylori* (60 patients) ranged between 25 and 65 years (±years). It was noticed that *H. pylori* infection increases significantly with age in most of cases, 45/60 (75%), aged> 45 years with *P* value <0.001. Genotypes *s*1*m*1 and *s*2*m*2 showed statistically significant association with patients aged >45 years (*P* value <0.001), while the association with *s*1*m*2 was insignificant (*P* > 0.05).

## 4. Discussion

*H. pylori* is recognized as the most prevalent infectious disease in human being. It causes persistent gastritis that is interrelated to PUD and gastric adenocarcinoma [1]. In all guidelines, *H. pylori* eradication is accepted as the primary priority strategy for preventing gastric cancer [17].

Dyspeptic patients are more likely to have *H. pylori* infection than asymptomatic individuals. The burden of *H. pylori* infection in our patients with dyspepsia was high; about two-thirds of patients tested positive for RUT. Our findings were close to those of an Egyptian study in Mansoura [18]. The prevalence of *H. pylori* infection in different countries is variable. For instance, it is more than 80% in many areas such as Japan, South America, Turkey, and Pakistan, while in England it is lower (20%) [19].

In this study, antral gastritis was the predominant endoscopic finding, representing 36.6%. Moreover, antral gastritis, diffuse erosive gastroduodenitis, duodenal ulcer, gastric ulcer, and combined duodenal and gastric ulcer showed statistically significant association with RUT (*P* < 0.001, <0.001, 0.03, 0.017, and 0.04, respectively). Our findings agreed with many studies which reported that most of *H. pylori* patients developed acute gastritis that resolved spontaneously [20]. We noticed that 38.6% (58/150) of our patients were suffering from PUD with 51.7% (30/58) of them having *H. pylori.* This agrees with a Kenyan study, which reported that 30% had PUD with *H. pylori* infection [21].

*H. pylori cagA* is a highly immunogenic protein encoded by *cagA* gene. It is associated with cell injury and more severe clinical outcomes, including duodenal ulcer and gastric adenocarcinoma [22]. *vacA* disrupts epithelial cell tight junctions, alters the host inflammatory response, and suppresses T cell activation [16].

The relation of the *vacA* and *cagA* genotypes with clinical outcomes represented by endoscopic findings was examined. About two-thirds (66%) of *H. pylori* positive specimens had two or at least one of the virulence genes examined in our study. *vacA* and *cagA* genotypes were detected in 61.6% and 53%, respectively, of the 60 *H. pylori* strains. Similarly, a previous study on Cuban patients detected *vacA* gene in 61.6% of the studied *H. pylori* strains, considering it the main virulent gene in most of the strains [23]. Another study in Algeria identified the *cagA* gene in 58% patients [24], and the percentages were as follows in other countries: in Pakistan, 24.2% [25], and, in Japan, 90%, which is correlated with the increased prevalence of gastric cancer in that country [15]. Because of the high degree of geographic variability of *H. pylori*, certain *H. pylori* genotypes are possibly associated with severe clinical outcomes in some countries, while presenting as less harmful or even harmless variants in other regions. The observed discrepancies in different studies regarding *H. pylori* virulence genes may be attributed to difference in facilities or the limitations of PCR methods.

Regarding *cagA* and *VacA* genotypes, we found that *vacA* *+* *cagA*+/*s*1*m*1 was the most predominant (26/60, 43.3%). Similarly, *s*1*m*1 genotype is the most prevalent genotype in Asian population [26]. In our series, we noticed that *H. pylori* infected patients with PUD, gastritis, and GERD (50%, 41.6%, and 25%, respectively) had a statistically significant association with *cag*A genotype (*P* < 0.001). This agrees with a Saudi study which confirmed the association between *vacA* genotype and severe gastric endoscopic findings [27].

However, *cagA*+ was not found in our study as a single genotype. It was rather linked to *vacA s*1*m*1 in (26/60), *vacA s*1*m*2 (5/60), or *s*2*m*2 (1/60).

In this study, a lower percentage of *vacA s*2*m*2 genotype was noticed, which is considered as a less virulent form as compared with the acutely damaging *vacA s*1*m*1 as stated by Falsafi and his colleagues [28]. In our studied population, *vacA*+/*cagA*− genotype *s*1*m*1 was the least prevalent, and this is consistent with an Iranian study that was carried out in 2015 [28].

## 5. Conclusion

*Helicobacter pylori* infection is prevalent among dyspeptic patients. The main genotype combinations in our studied population were *vacA*+/*cagA*+ of *s*1*m*1 genotype and they were frequently associated with gastritis and GERD, while *vacA*−/*cagA*− patients presented mainly with gastritis. The less virulent (*s*2*m*2) genotypes were found in *vacA*+/*cagA*− and *vac*A*+*/*cagA*+ patients. Eventually, the advanced molecular methods are recently used as dependable tools for characterization of *H. pylori* virulent strains because of their increasing sensitivity and specificity.

### 5.1. Main Points

- *Helicobacter pylori* infection is prevalent among dyspeptic patients reflecting the increased risk of gastric disorders including gastric carcinoma.
- Knowledge of *H. pylori* virulence genes can be of clinical significance through improving the clinical prediction of disease risk and identifying those in need to more surveillance and eradication of the infection to prevent serious consequences.
- Advanced molecular methods can be used as dependable tools for characterization of *H. pylori* virulent strains because of their increasing sensitivity and specificity.

## Figures and Tables

**Table 1:** Primers used for the amplification of *vacA* alleles and *cagA*.

Region amplified	Primers	Primers sequence	Amplicon size	Ref
*vacA s*1/*vacA s*2	VAI-F	5′-ATGGAAATACAACAAACACAC-3′	259/286	Ito et al. [15]
	VAI-R	5′-CTGCTTGAATGCGCCAAAC-3′		
*vacA m*1/*vacA m*2	VAG-F	5′-CAATCTGTCCAATCAAGCGAG-3′	567/642	Jones et al. [7]
	VAG-R	5′-GCGTCAAAATAATTCCAAGG-3′		
*cagA*	*cagA*5c-F	5′-GTTGATAACGCTGTCGCTTC-3′	350	Graham and Yamaoka [16]
	*cagA*3c-R	5′-GGGTTGTATGATATTTTCCATAA-3′		

**Table 2:** Endoscopic findings in relation to RUT and PCR.

Findings	RUT^*∗∗*^ (100)	*P* value	OR (95% CI)	PCR^*∗∗∗*^ (60)	*P* value	OR (95% CI)
Antral gastritis (55)	55 (55%)	<0.001^*∗*^		20 (33.3%)	0.489	0.8 (0.4–1.6)
Gerd (34)	36 (36%)	0.465	1.3 (0.6–2.7)	15 (25%)	0.057	0.5 (0.2–1.0)
Pangastritis (40)	30 (30%)	0.192	1.7 (0.8–3.9)	5 (8.3%)	<0.001^*∗*^	0.1 (0.1–0.4)
Diffuse gastric mucosal nodularity (40)	20 (20%)	0.172	0.6 (0.3–1.3)	10 (16.6%)	0.115	0.5 (0.2–1.2)
Diffuse erosive gastroduodenitis (34)	15 (15%)	<0.001^*∗*^	0.3 (0.1–0.6)	8 (13.3%)	<0.001^*∗*^	0.1 (0.1–0.3)
Duodenal ulcer (30)	25 (25%)	0.030^*∗*^	3 (1.1–8.4)	17 (28.3%)	0.084	0.5 (0.3–1.1)
Gastric ulcer (20)	18 (18%)	0.017^*∗*^	5.3 (1.2–23.7)	8 (13.3%)	<0.001^*∗*^	0.1 (0.1–0.3)
Combined duodenal and gastric ulcers (8)	8 (8%)	0.040^*∗*^		5 (8.3%)	<0.001^*∗*^	0.1 (0.0–0.2)
Hiatus hernia (3)	0 (0%)	0.014^*∗*^		0 (0%)	<0.001^*∗*^	
Gastric cancer (3)	2 (2%)	1.000	1 (0.1–11.3)	0 (0%)	<0.001^*∗*^	
Duodenal diverticulum (2)	0 (0%)			0 (0%)	<0.001^*∗*^	
Gastric polyp (1)	0 (0%)	0.156		0 (0%)	<0.001^*∗*^	
Barrette esophagus (1)	0 (0%)	0.156		0 (0%)	<0.001^*∗*^	
Normal mucosa (4)	0 (0%)	0.156		0 (0%)	<0.001^*∗*^	

^*∗*^ *P* < 0.001: highly statistically significant; ^*∗∗*^RUT: rapid urease test; ^*∗∗∗*^PCR: polymerase chain reaction.

**Table 3:** Association between virulence genes and endoscopic findings in *H. pylori* strains (no. = 60).

Virulence genes	*vacA* genotypes (No.)	Endoscopic findings (*n* = 60)							
		PUD^*∗*^ (30) No. (%)	*P* value	Gastritis (25) No. (%)	*P* value	GERD^*∗∗*^ (15) No. (%)	*P* value	Others (18) No. (%)	*P* value
*vacA*+/*cagA*+	*s*1*m*1 (26)	10 (33.3%)	<0.001^*∗*^	12 (20%)	<0.001^*∗*^	8 (13.3%)	<0.001^*∗*^	6 (10%)	<0.001^*∗*^
	*s*1*m*2 (5)	5 (16.6%)		4 (6.6%)		2 (3.3%)		0 (0.0%)	
	*s*2*m*2 (1)	1 (3.3%)		0 (0.0%)		1 (1.6%)		0 (0.0%)	
*vacA*+/*cagA*−	*s*1*m*1 (1)	0 (0.0%)	0.392	1 (3.3%)	0.392	0 (0.0%)		0 (0.0%)	
	*s*1*m*2 (2)	1 (3.3%)		1 (3.3%)		0 (0.0%)		0 (0.0%)	
	*s*2*m*2 (2)	0 (0.0%)		2 (6.6%)		0 (0.0%)		0 (0.0%)	
*vacA*−/*cagA*−		13 (23.3%)		5 (11.6%)		4 (1.6%)		12 (16.6%)	

^*∗*^PUD: Peptic Ulcer Disease. ^*∗∗*^GERD: Gastroesophageal Reflux Disease.

## Data Availability

The data supporting the findings of this study are available within the article.
